# The Influence of Materials, Heterostructure, and Orientation for Nanohybrids on Photocatalytic Activity

**DOI:** 10.1186/s11671-019-2851-z

**Published:** 2019-01-14

**Authors:** Lidan Wang, Zisheng Su, Junsheng Yuan

**Affiliations:** 1 College of Chemical Engineering and Material, Quanzhou Normal University, Quanzhou, 362000 Fujian China; 2 College of Physics and Information Engineering, Quanzhou Normal University, Quanzhou, 362000 Fujian China

**Keywords:** Nanohybrids, Electrodeposition, Heterostructure, Photocatalytic

## Abstract

In this work, different structures based on electrodeposited n-type ZnO nanorods and p-type Cu_2_O, CuSCN, and NiO nanostructures are fabricated for the degradation of methyl orange (MO). The influence of materials, heterostructure, and orientation for nanohybrids on photocatalytic activity is discussed for the first time. The heterojunction structures show remarkable enhancement compared to the bare semiconductor. The morphology of nanostructure has mainly an influence on the photocatalytic activity. NiO has the highest catalytic activity among the four pristine semiconductor nanostructures of ZnO, Cu_2_O, CuSCN, and NiO. The greatest enhancement of the photocatalytic activity is obtained using a ZnO/NiO (1 min) heterostructure attributed to the heterojunction structure and extremely higher specific surface area, which can degrade MO (20 mg/L) into colorless within 20 min with the fastest photocatalytic speed among homogeneous heterojunction structures. Meanwhile, the methodology and data analysis described herein will serve as an effective approach for the design of hybrid nanostructures for solar energy application, and the appropriate nanohybrids will have significant potential to solve the environment and energy issues.

**Electronic supplementary material:**

The online version of this article (10.1186/s11671-019-2851-z) contains supplementary material, which is available to authorized users.

## Background

Hybrid nanomaterials with outstanding optical, electronic, and magnetic properties have attracted much interest in recent years due to their widespread applications in environment remediation [1, 2] and solar energy conversion [3, 4]. In recent years, several varieties of nanohybrids have been developed, for example, graphene oxide nanocomposites [5], TiO_2_/BiVO_4_ nanocomposites [6], 3D-printed hydrogel nanocomposites [7], and Ru/Li_2_O nanocomposites [8]. Among the different kinds of nanomaterials, heterojunctions based on different nano-semiconductors have developed into an important research area due to their attractive photocatalytic [9–11] and the photovoltaic [12–14] properties. Recently, several research works have been done on various catalytic applications of dye degradation, such as nanohybrid membrane organocatalyst [15], bioinorganic nanohybrid catalyst [16], and green nanohybrid catalyst [17]. Due to the toxicity, the organic dyes in the wastewater pose a serious threat to human health [18, 19]. Thus, the conversion of organic dyes into harmless substances is essential for human life and sustainable development. A variety of inorganic semiconductor materials with different morphologies have been explored as photocatalysts for wastewater purification under UV or visible-light irradiation [20–22], especially zinc oxide (ZnO) and titanium dioxide (TiO_2_) one-dimensional (1D) nanomaterials. So far, ZnO nanomaterials obtained the most widely investigation among various semiconductors, which could be attributed to their efficient electron transfer performance [23], providing photogenerated holes for strong oxidation, better environmental friendly feature, non-toxicity, low cost, and good stability and being widespread in the earth [24, 25]. However, the photocatalytic activity of ZnO is severely limited by its internal defects: narrower UV-visible region response due to its large bandgap and high eventuality of recombination of photogenerated electron-hole pairs [26, 27]. In order to overcome these limitations, numerous measures have been taken, such as doping [28], composite noble metals, such as Au [29, 30] and Ag [31]; and combining with other semiconductors, such as CdS [32], ZnSe [33], CdSe [34, 35], and PbS [36, 37]. Obtaining heterojunctions based on ZnO and other semiconductors has been proved to be a feasible way to improve visible light response and wastewater degradation efficiency. Recently, several heterojunctions based on ZnO nanomaterial and p-type nano-semiconductor on degradation have been developed. Recently, the Cu_2_O-ZnO heterostructures on photocatalysis were reported by Wang et al. [38] and Yu et al. [39]. Luo and coworkers reported the ZnO/CNF/NiO heteroarchitecture for high-performance photocatalysis [40]. Liu et al. reported electrospun nanofiber NiO/ZnO heterojunctions with enhanced photocatalytic activity [41]. ZnO/CdS structure also has higher photocatalytic activity than pristine materials [42]. These reports indicate that the heterostructures possessed higher photocatalytic activity of dye decomposition than pristine semiconductors. However, the efficiency of photocatalytic degradation of methyl orange (MO) needs further improvement. Also, the design of the heterojunction structure needs further investigation, for instance, reducing the cost by getting rid of noble metal and using a simple method such as electrodeposition and lower reaction temperature. In this study, ZnO, Cu_2_O, CuSCN, and NiO nanostructures are prepared by a low-cost, simple electrodeposition method at room temperature. Heterojunction structures of different materials and different orientation are fabricated based on n-type ZnO nanorods and p-type Cu_2_O, CuSCN, and NiO nanostructures. The heterostructures exhibit much better photocatalytic performance for photocatalytic degradation of MO than pristine n-type material or p-type material. The influence on the orientation of the heterojunction is dependent on the crystal quality of the upper material of the heterojunction. The influence on the material of different reaction conditions is dependent on the morphology and quality of the nanostructures. Among the three p-type materials used in our work, NiO has the most excellent photocatalytic performance. The ZnO/NiO (1 min) can decompose the MO (20 mg/L) aqueous solution from orange to colorless within 20 min. It reveals that the material and the orientation can both give an effect on the photocatalytic performance, which has great importance meaning for the decomposition of organic pollutants; moreover, this study is the first thorough study of the influence of materials, orientation, and heterostructure on photocatalytic activity and may promote further investigations on more nanohybrids to obtain higher photocatalytic efficiency.

## Methods

### Experimental Materials

Indium tin oxide (ITO)-coated glass (CSG Holding Co., Ltd., 15 Ω/sq), zinc nitrate (Zn(NO_3_)·6H_2_O), hexamethylenetetramine (HMT), copper (II) sulfate pentahydrate, sodium hydroxide, lactic acid, potassium thiocyanate, ethylenediaminetetraacetic acid, triethanolamine, and nickel nitrate hexahydrate are all purchased from Sinopharm Chemical Reagent Co., Ltd. All these materials are of analytical grade and used as received without further purification.

### Preparation of Nanostructures

The cost-effective electrodeposition method is used in this work for the preparation of the large area of nanostructures because of the low-temperature processing, arbitrary substrate shapes, and precise control of the size of nanostructures [43]. All depositions are carried out in a configured glass cell in which an ITO substrate, a platinum plate, and an Ag/AgCl electrode in saturated KCl or saturated calomel electrode (SCE) serve as the working electrode, the counter electrode, and the reference electrode, respectively. The detailed reaction conditions for the fabrication of all the nanostructures by electrodeposition are shown in Table 1. The pH value of Cu_2_O reaction solution is regulated from 10 to 12 by NaOH. The pH value of CuSCN reaction solution is around 1.5. Finally, all the above deposited samples are rinsed with deionized water to remove the electrolyte and dried in air naturally. No post deposition annealing is employed.

**Table 1:** Reaction conditions for the fabrication of the nanostructures by electrodeposition

Nanostructures	Reaction precursors	Temperature (°C)	Deposition time (min)	Reference electrode and potential
ZnO nanorods	0.005 M Zn(NO_3_)_2_ 0.005 M HMT	90	20	Ag/AgCl − 0.9 V
Cu_2_O nanostructures	0.5 M CuSO_4_ 3 M lactic acid	60	20–40	Ag/AgCl − 0.3 V
CuSCN nanowires	0.012 M CuSO_4_ 0.012 M EDTA 0.003 M KSCN	Room temperature	5	SCE − 0.4 V
CuSCN 3D nanostructures	1 M CuSO_4_ 1 M EDTA 1 M KSCN	Room temperature	10	Ag/AgCl − 0.3 V
NiO nanostructures	0.2 M Ni (NO_3_)_2_ 0.2 M HMT	50	1–10	Ag/AgCl − 2.2 V

### Characterizations

X-ray diffraction (XRD) patterns are measured with a Rigaku D/Max-2500 diffractometer using Cu Kα radiation (*λ* = 1.54 Å) at room temperature. The scan rate is 10°/min. The voltage and current are 40 kV and 40 mA. Surface and cross-sectioned structure of the samples are characterized by scanning electron microscopy (SEM) using a Philips-FEI XL 30-SFEG at room temperature without any surface coating. The accelerating voltage is 10–20 kV. The optical properties of the samples are investigated by UV-vis diffuse reflectance spectrometry (UV-vis DRS) using a Shimadzu UV-3101PC UV-vis spectrophotometer at room temperature.

### Photocatalytic Decomposition Experiments

Photocatalytic activities of prepared samples are evaluated towards the degradation of MO in aqueous solution. A 500-W Xe lamp is a light source of photocatalytic reaction devices. In a photodegradation process, the sample is placed into a quartz reactor filled with 3 mL of MO (20 mg/L) aqueous solution. Before irradiation, the aqueous solution is kept in the dark for 60 min to reach the adsorption equilibrium of MO. After a specific irradiation time, the photocatalytic decomposition performance is analyzed by measuring the absorbance of MO solution at its characteristic wavelength (465 nm) with a UV-vis spectrophotometer. All of the samples are performed in independent experiments and carried out at room temperature.

## Results and Discussion

### Preparation of Nanostructures and Composition Analysis

**Fig. 1:**
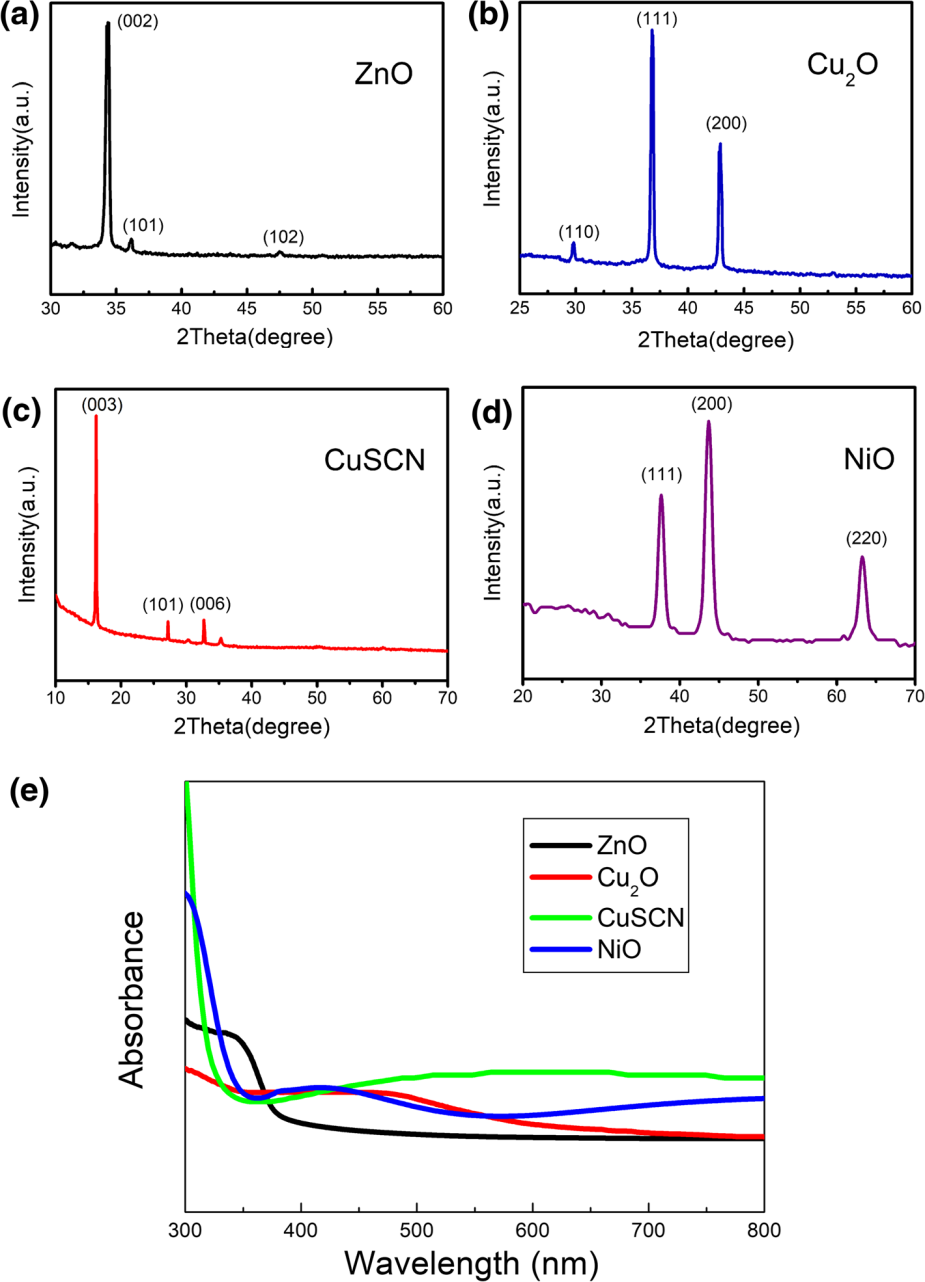
X-ray photoelectron spectra of ZnO (**a**), Cu_2_O (pH 10, 20 min) (**b**), CuSCN (3D) (**c**), and NiO (1 min) (**d**) nanostructures prepared by electrodeposition method and the absorbance spectra (**e**) of ZnO, Cu_2_O (pH 10, 20 min), CuSCN(3D), and NiO(1 min) nanostructures prepared by electrodeposition method

All the ZnO, Cu_2_O, CuSCN, and NiO nanostructures are synthesized by the cost-effective electrodeposition method at room temperature. The electrodeposition is performed in a standard three-electrode electrochemical cell in the potentiostatic mode. The crystal textures of ZnO, Cu_2_O, CuSCN, and NiO nanostructures are characterized via XRD profiles. The XRD images of ZnO, Cu_2_O, CuSCN, and NiO nanostructures prepared by the electrodeposition method are shown in Fig. 1. A set of peaks in Fig. 1a appear at 2*θ* of ca. 34.36°, 36.12°, and 47.48° for the ZnO nanorods, which are assigned to the (002), (101), and (102) of ZnO crystals, respectively. All the peaks in the ZnO nanorods can be indexed to the hexagonal wurtzite structure of ZnO, and no other detectable phases exist in the ZnO nanostructures, which are similar as XRD profiles in Ref. [39]. Moreover, the strong ZnO (002) peak indicates that oriented nanorods with high crystallinity are obtained. Three peaks in Fig. 1b at 2*θ* of ca. 29.78°, 36.81°, and 42.89° are observed for the electrodeposited Cu_2_O film on ITO substrate, which are assigned to the (110), (111), and (200) of Cu_2_O crystals, respectively, indicating that Cu_2_O has the pure cupric cubic structure with a (111) preferred orientation, which is the same as XRD profiles in Ref. [38]. The diffraction of peaks in Fig. 1c appears at 2*θ* of ca.16.21°, 27.20°, and 32.69° and can be assigned to the (003), (101), and (006) planes of CuSCN crystals, respectively, which can be indexed to a rhombohedral structure *β*-CuSCN [44]. The XRD patterns in Fig. 1d are assigned to the three main NiO peaks at 37.52°, 43.26°, and 62.86°, which refer to the planes (111), (200), and (220), respectively, as similar as XRD profiles in Ref. [39]. All the XRD patterns reveal that none of the other phases are detected, and the nanostructures are without impurity. Figure 1 e shows the absorbance spectra of ZnO, Cu_2_O, CuSCN, and NiO nanostructures prepared by the electrodeposition method. As shown in Fig. 1e, ZnO nanorods can only absorb the high-energy light with the wavelength shorter than 370 nm. An absorbance band edge at 600 nm can be observed for Cu_2_O, as shown in Fig. 1e, which is consistent with the band gap of Cu_2_O (2.1 eV). As shown in Fig. 1e, CuSCN has a low and broad absorption with the wavelength longer than 350 nm and NiO has an absorption between 350 and 500 nm but a low absorption with the wavelength longer than 500 nm. All the absorption of ZnO, Cu_2_O, CuSCN, and NiO nanostructures is in the ultraviolet and visible range, and this will guarantee the absorption of the ultraviolet light under Xe lamp irradiation in the photocatalytic decomposition experiments and the consequent generating of electron-hole pairs.

### Design and Morphology of the Heterostructures

**Fig. 2:**
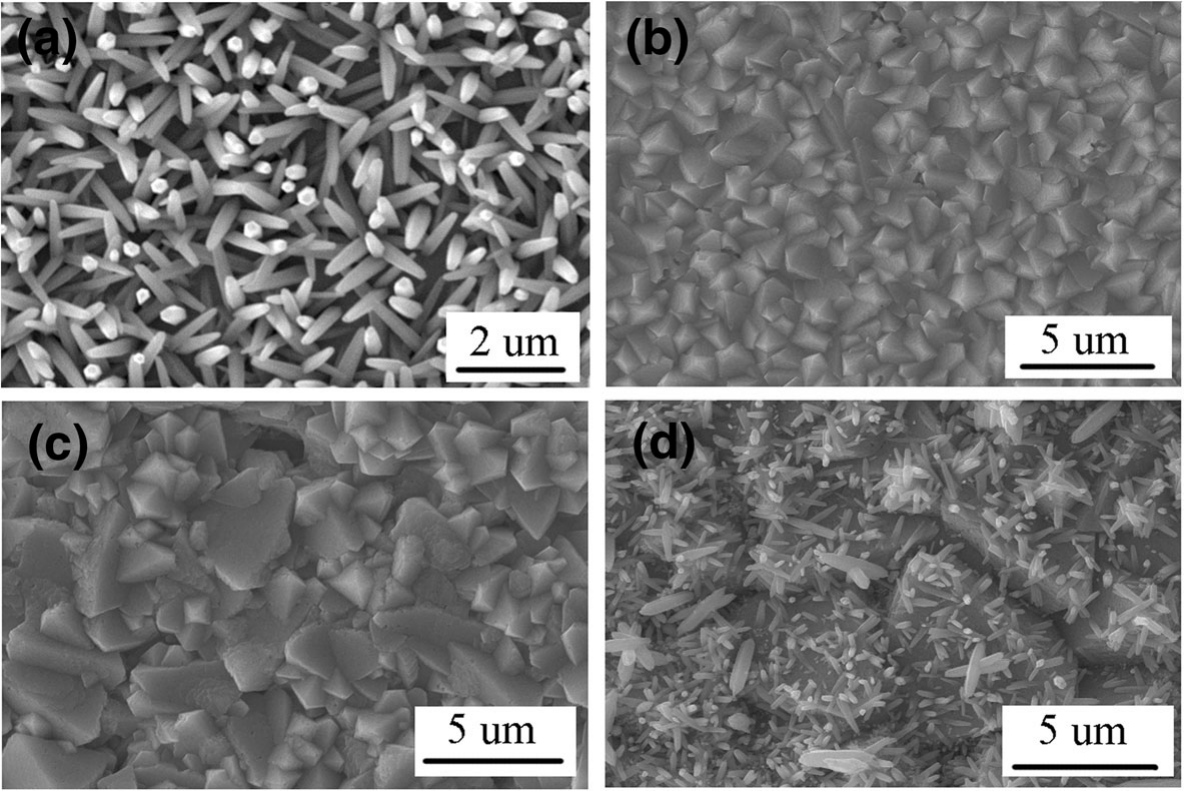
The top view SEM image of ZnO nanorods (**a**), ZnO/Cu_2_O (pH 12, 20 min) heterojunction (**b**), Cu_2_O (pH 12, 20 min) (**c**), and Cu_2_O (pH 12, 20 min)/ZnO heterojunction (**d**)

**Fig. 3:**
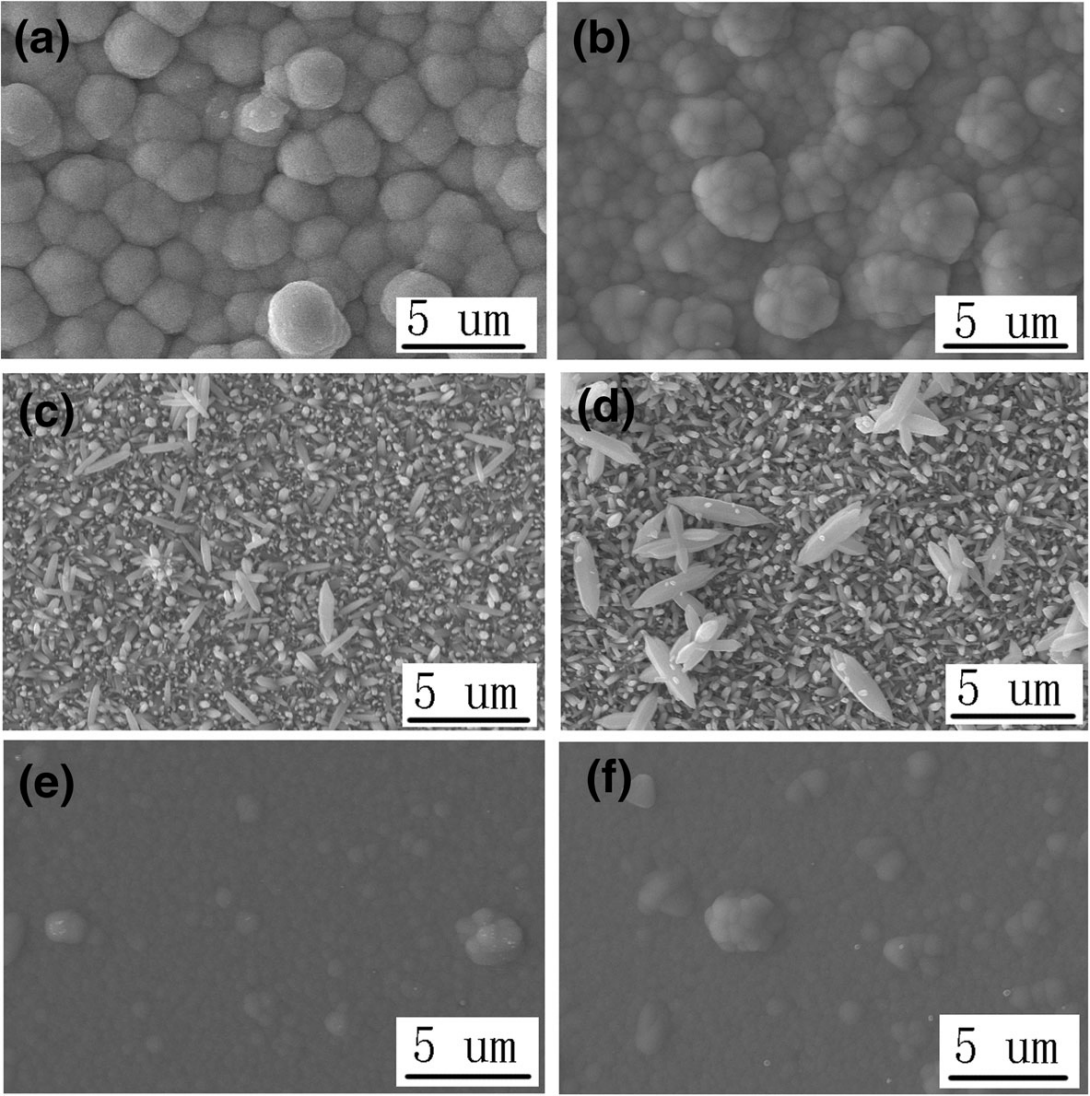
The top view SEM image of ZnO/Cu_2_O (pH 10, 20 min) heterojunction (**a**), ZnO/Cu_2_O (pH 10, 40 min) heterojunction (**b**), Cu_2_O (pH 10, 20 min)/ZnO heterojunction (**c**), Cu_2_O (pH 10, 40 min)/ZnO heterojunction (**d**), Cu_2_O (pH 10, 20 min) (**e**), and Cu_2_O (pH 10, 40 min) (**f**)

Different heterojunctions based on n-type ZnO nanorods and p-type Cu_2_O, CuSCN, and NiO nanostructures with different orientation are fabricated. Firstly, the ZnO, Cu_2_O, ZnO/Cu_2_O, and Cu_2_O/ZnO are prepared for photocatalytic degradation of MO. Figure 2 shows the top view SEM image of ZnO nanorods (a), ZnO/Cu_2_O (pH 12, 20 min) heterojunction (b), Cu_2_O (pH 12, 20 min) (c), and Cu_2_O (pH 12, 20 min)/ZnO heterojunction (d). Additional file 1: Figure S1 shows the cross-sectional view of these four structures. Figure 3 shows the top view SEM image of ZnO/Cu_2_O (pH 10, 20 min) heterojunction (a), ZnO/Cu_2_O (pH 10, 40 min) heterojunction (b), Cu_2_O (pH 10, 20 min)/ZnO heterojunction (c), Cu_2_O (pH 10, 40 min)/ZnO heterojunction (d), Cu_2_O (pH 10, 20 min) (e), and Cu_2_O (pH 10, 40 min) (f). Additional file 1: Figure S2 shows the cross-sectional view of these six structures. As seen from the top view shown in Fig. 2a and the cross-sectional view in Additional file 1: Figure S1(a), the ZnO nanorods obtained by the electrodeposition method are almost the structure of hexagon prism. The diameter and the length of the nanorods are in the ranges of 200–300 nm and 800–1200 nm, respectively. As shown in Figs. 2c and 3e and f, it can be noted that the Cu_2_O crystals grow from cubes to octahedras when the pH value of the electrodeposition solution alters from 10 to 12. The crystals obtained at pH ~ 10 with the reaction time of 20 and 40 min are not all the perfect cubes due to the different dissolution of the crystal in the reaction solution [45]. It can also be clearly seen that the Cu_2_O crystals grow bigger and denser according to longer time, and the Cu_2_O crystals will crowd and aggregate together when the reaction time is longer. In the aggregating process, the Cu_2_O crystals will be crowded out of shape due to the great density. As shown in Figs. 2b and 3a and b, it can be concluded that Cu_2_O crystals grown on the ZnO nanorods are as compact as the Cu_2_O crystals grown on the bare ITO glass substrate and are some smaller than the Cu_2_O crystals grown on ITO glass due to the different nucleating point. When the pH value of the reaction solution is 10, the ZnO nanorods grown on the Cu_2_O crystals are more compact than the ZnO nanorods grown on ITO glass, and the diameter and the length of the ZnO nanorods grown on the Cu_2_O crystals are almost the same with the ZnO nanorods grown on ITO glass, as shown in Fig. 3c and d. The ZnO nanorods grown on the Cu_2_O crystals (pH 10, 40 min) are a little denser than the ZnO nanorods grown on the Cu_2_O crystals (pH 10, 20 min), and much larger ZnO rods will appear on the layer of the ZnO nanorods. As shown in Fig. 2d, the ZnO nanorods grown on the Cu_2_O crystals (pH 12, 20 min) are much sparser than the ZnO nanorods grown on the ITO glass. The diameter and the length of the ZnO nanorods grown on the Cu_2_O crystals (pH 12, 20 min) are not homogeneous in a large range.

**Fig. 4:**
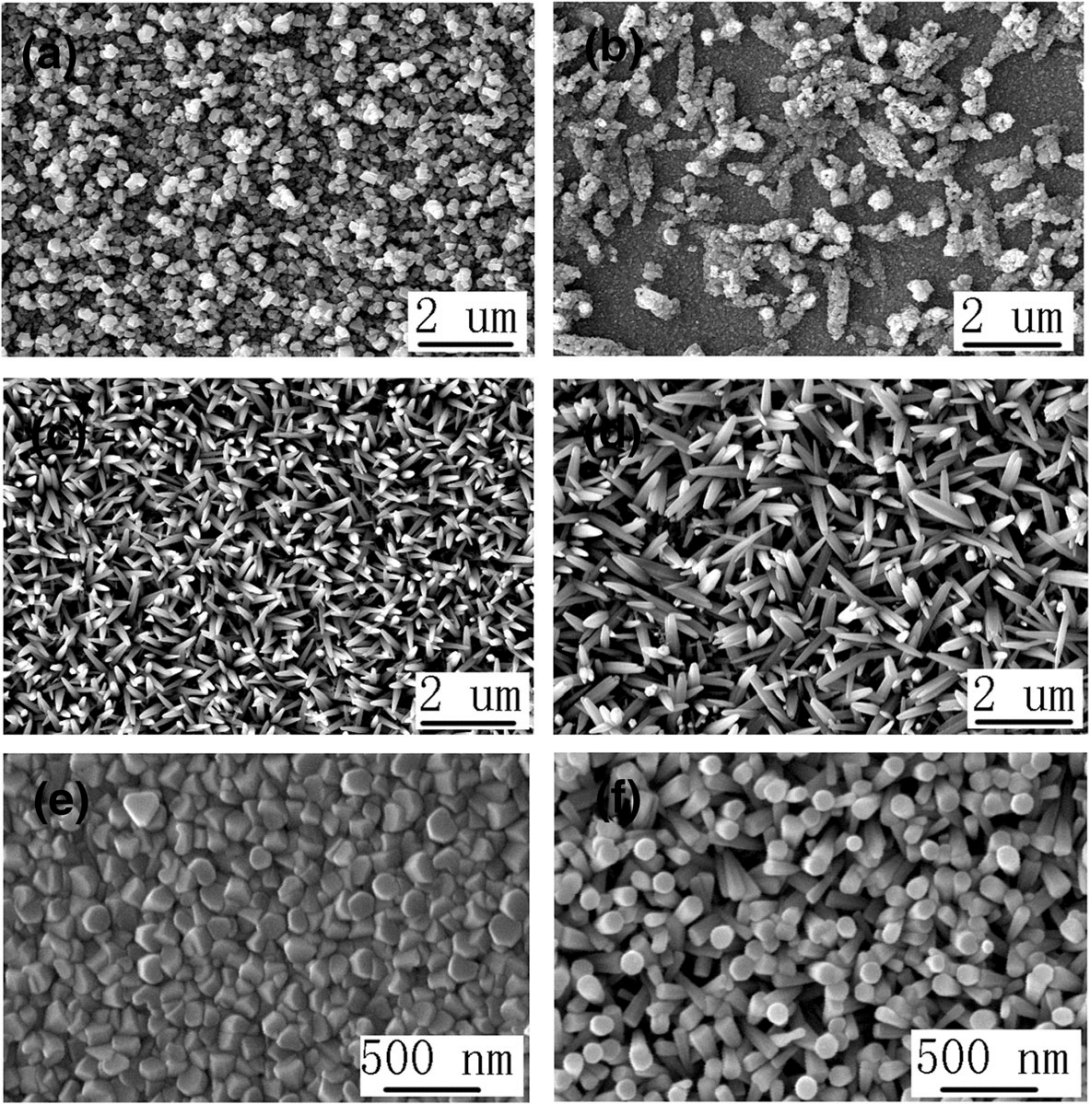
The top view SEM image of ZnO/ CuSCN (3D) heterojunction (**a**), ZnO/ CuSCN (NWs) heterojunction (**b**), CuSCN (3D)/ZnO heterojunction (**c**), CuSCN (NWs)/ZnO heterojunction (**d**), CuSCN (3D) (**e**), and CuSCN (NWs) (**f**)

Secondly, ZnO, CuSCN, ZnO/CuSCN, and CuSCN/ZnO are prepared for photocatalytic degradation of MO. Two different CuSCN nanostructures, hexagonal prism-like (3D) and nanowire (NW) structures, are prepared by the electrodepositon method. Figure 4 shows the top view SEM image of ZnO/CuSCN (3D) heterojunction (a), ZnO/CuSCN (NWs) heterojunction (b), CuSCN (3D)/ZnO heterojunction (c), CuSCN (NWs)/ZnO heterojunction (d), CuSCN (3D) (e), and CuSCN (NWs) (f). Additional file 1: Figure S3 shows the cross-sectional view of these six structures. The CuSCN (3D) and CuSCN (NWs) structures electrodeposited on ZnO nanorods are denser than the ones on ITO glass, as shown in Fig. 4a and b. The ZnO nanorods under CuSCN (3D) structures are partially etched by the CuSCN reaction solution with an erosive pH of 1.5, as shown in Fig. 4a and Additional file 1: Figure S3(a). The ZnO nanorods under CuSCN (NWs) structures are mainly etched by the CuSCN reaction solution with a pH of 1.5, but the outline of ZnO nanorods is maintained after the electrodeposition of CuSCN (NWs) structures, as shown in Fig. 4b and Additional file 1: Figure S3(b). The CuSCN (3D) on the ZnO nanorods is much more intensive than the CuSCN (NWs) structures on the ZnO nanorods, and the ZnO nanorods under CuSCN (NWs) structures almost disappear only remaining the vestige of ZnO hexagon prism. The ZnO nanorods prepared on the layer of CuSCN are more compact than the ZnO nanorods grown on ITO glass, and the diameter and the length of the ZnO nanorods grown on CuSCN are smaller than the ZnO nanorods grown on ITO glass due to the different nucleating point, as shown in Fig. 4c and d. The CuSCN (3D) and CuSCN (NWs) structures prepared by the electrodeposition method on ITO glass are oriented with highly dense and almost vertical to the substrate with the diameter of about 100 nm and 80 nm, respectively, as shown in Fig. 4e and f.

**Fig. 5:**
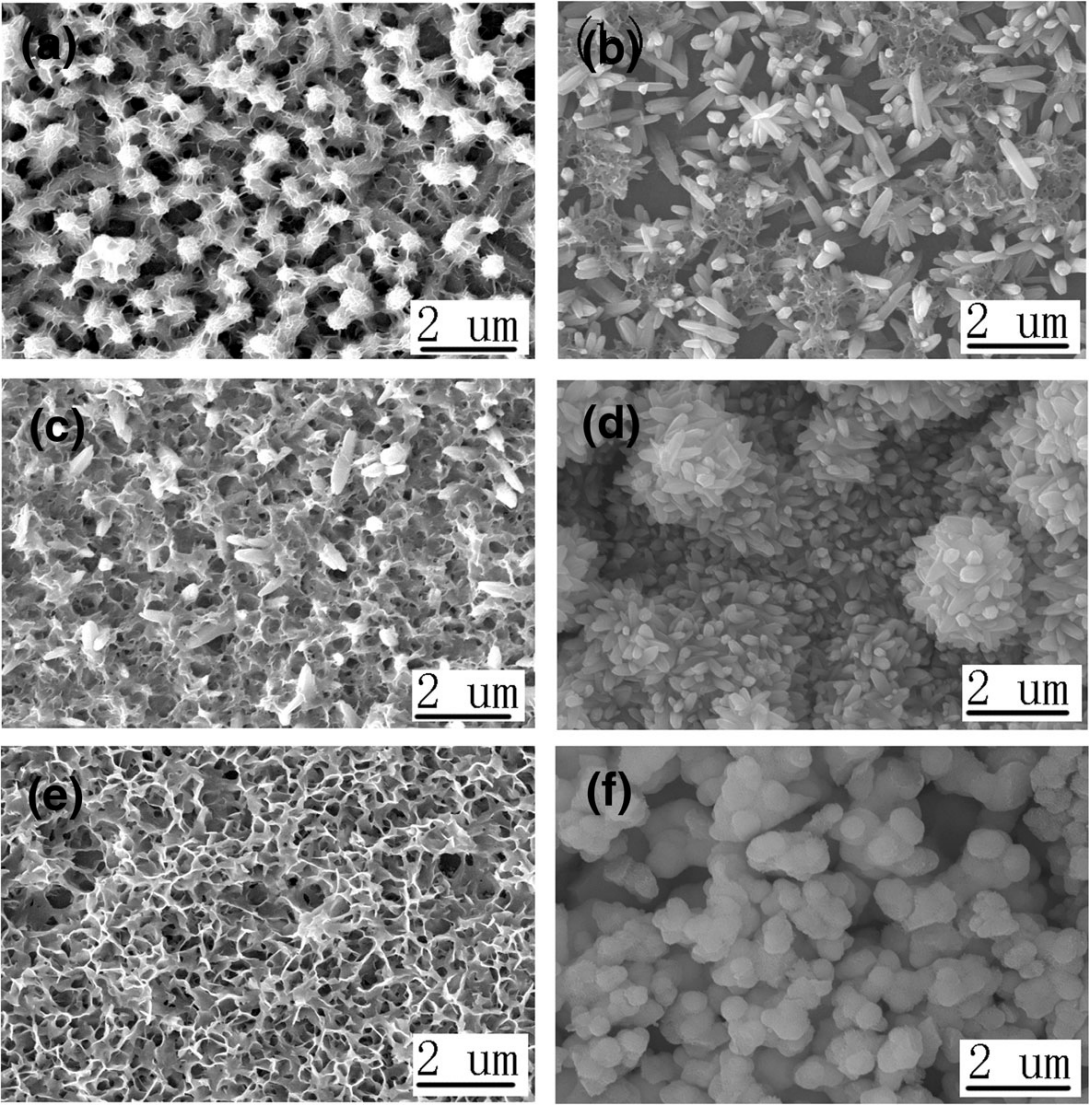
The top view SEM image of ZnO/NiO (1 min) heterojunction (**a**), ZnO/NiO (10 min) heterojunction (**b**), NiO (1 min)/ZnO heterojunction (**c**), NiO (10 min)/ZnO heterojunction (**d**), NiO (1 min) (**e**), and NiO (10 min) (**f**)

Finally, the ZnO, NiO, ZnO/NiO, and NiO/ZnO are prepared for photocatalytic degradation of MO. Figure 5 shows the top view SEM image of ZnO/NiO (1 min) heterojunction (a), ZnO/NiO (10 min) heterojunction (b), NiO (1 min)/ZnO heterojunction (c), NiO (10 min)/ZnO heterojunction (d), NiO (1 min) (e), and NiO (10 min) (f). Additional file 1: Figure S4 shows the cross-sectional view of these six structures. The NiO nanostructure electrodeposited on ZnO nanorods for 1 min is meshwork intersected with ZnO nanorods, as shown in Fig. 5a and Additional file 1: Figure S4(a). The ZnO nanorods electrodeposited on the NiO nanostructure (1 min) are partially exposed growing through the meshwork of NiO (1 min), and the remnant part of the ZnO nanorods are remained in the meshwork which cannot be seen in the top view SEM images, as shown in Fig. 5c and Additional file 1: Figure S4(c). The NiO nanostructure electrodeposited on ITO glass for 1 min is the multi-layer interspersed meshwork uniformly distributed on the ITO glass with high specific surface area, as shown in Fig. 5e and Additional file 1: Figure S4(e). The NiO nanostructure electrodeposited on ZnO nanorods for 10 min is flowers consisted of many particles, as shown in Fig. 5b and Additional file 1: Figure S4(b). The NiO nanostructure electrodeposited on ITO glass for 10 min is consisted of many NiO particles which can form a compact layer on the ITO glass and a particle layer on the compact layer, as shown in Fig. 5f and Additional file 1: Figure S4(f). From the cross-sectional view of SEM in Additional file 1: Figure S4(f), some cracks can be found in the compact layer on the ITO glass due to the extrusion force generated by NiO particles. When ZnO is electrodeposited on NiO (10 min), the smaller ZnO nanorods (compared to the ones on the ITO glass) grow on the NiO particle layer, and the shape of the NiO particles disappears only remaining the morphology of the ZnO nanorods, as shown in Fig. 5d and Additional file 1: Figure S4(d). The cracks in the compact layer of NiO on the ITO glass can also be seen in Additional file 1: Figure S4(d), and some cracks in the structure of ZnO nanorods are generated by the cracks in the compact layer.

### Photocatalytic Activity

The photocatalytic properties are investigated via the degradation of MO, a common organic pollutant [46, 47]. Three systems of ZnO/Cu_2_O, ZnO/CuSCN, and ZnO/NiO are discussed including pristine ZnO, pristine Cu_2_O (or CuSCN or NiO), ZnO/Cu_2_O (or CuSCN or NiO), and Cu_2_O (or CuSCN or NiO) with four structures in every system. Although the electrodeposition method used in this work for the preparation of the nanostructures is green and environmental, the preparation method may require a considerable amount of undesirable chemicals which undermines the greenness of the methods. The problem such as the sustainable membrane recovery of chemicals can be solved by a continuous hybrid process comprising of a flow reactor and a subsequent nanofiltration unit for in situ solvent and reagent recycle which was developed by Szekely et al. [48]. Additional file 1: Figure S5 and Figure S6 exhibit the concentration changes of MO in the absence and in the presence of different photocatalysts. Obviously, the MO content has little change in the absence of the catalyst as compared to the addition of the catalyst. Under visible light irradiation, only 15% of MO is decomposed after 40 min in the absence of the catalyst, as shown in Additional file 1: Figure S5(a). Pristine ZnO nanorods exhibit a certain degree of photocatalytic activity for the decomposition of MO. However, due to the limitations of internal defects (large band gap and easy recombination of electron-hole pairs) and specific surface area, the photocatalytic performance is still poor, as shown in Additional file 1: Figure S6(a), Figure S6(b) and Figure S6(c). The comparison of the specific surface area of the nanostructures is conjectured from the size and density of the surface by SEM image [49–51]. The concentration changes of MO with ZnO, Cu_2_O (pH 10, 20 min), Cu_2_O (pH 10, 40 min), Cu_2_O (pH 12, 20 min), ZnO/Cu_2_O (pH 10, 20 min), ZnO/Cu_2_O (pH 10, 40 min), ZnO/Cu_2_O (pH 12, 20 min), Cu_2_O (pH 10, 20 min)/ZnO, Cu_2_O(pH 10, 40 min)/ZnO, and Cu_2_O (pH 12, 20 min)/ZnO as the catalysts are shown in Additional file 1: Figure S6(a). The intensity of the absorption peak is gradually diminished and blue-shifted as the irradiation time increased from 0 to 40 min. The blue shift can be attributed to dealkylation [52]. The photocatalytic performance of three different Cu_2_O, Cu_2_O (pH 10, 20 min), Cu_2_O (pH 10, 40 min), and Cu_2_O (pH 12, 20 min), is similar due to the similar morphology and the specific surface area, as shown in Figs. 2c and 3a and b. The photocatalytic performance of pristine Cu_2_O is poorer than that of pristine ZnO nanorods due to bigger crystal boundary, lower carrier mobility, smaller specific surface area, and easier recombination of electrons and holes. The photocatalytic performance of ZnO/Cu_2_O (pH 10, 20 min), ZnO/Cu_2_O (pH 10, 40 min), and ZnO/Cu_2_O (pH 12, 20 min) is almost the same owing to the similar morphology and the specific surface area of the upper layer Cu_2_O, as shown in Figs. 2b and 3e and f. The photocatalytic performance of three heterojunctions of ZnO/Cu_2_O is lower than that of pristine ZnO nanorods due to the smaller specific surface area of the upper layer Cu_2_O as a direct connect to the MO solution but is higher than that of pristine Cu_2_O owing to the effect of heterojunction of ZnO and Cu_2_O. The photocatalytic performance of Cu_2_O/ZnO architecture is the highest in the ZnO/Cu_2_O system due to their heterojunction structure and larger specific surface area of the upper layer ZnO. Compared to the sparse ZnO nanorods on Cu_2_O (pH 12, 20 min) and too much large ZnO nanorods on Cu_2_O (pH 10, 40 min), Cu_2_O (pH 10, 20 min)/ZnO has the best photocatalytic performance among three Cu_2_O/ZnO architectures as a result of perfect ZnO nanostructures grown on Cu_2_O (pH 10, 20 min), as shown in Figs. 2d and 3c and d. The influence on reaction pH, reaction time, and orientation of the heterojunction is discussed, and in conclusion, the reaction time has little effect on the photocatalytic performance in the ZnO/Cu_2_O system. In summary, in the ZnO/Cu_2_O system, Cu_2_O (pH 10, 20 min)/ZnO has the best photocatalytic performance.

The concentration changes of MO with ZnO, CuSCN (3D), CuSCN (NWs), ZnO/CuSCN (3D), ZnO/CuSCN (NWs), CuSCN (3D)/ZnO, and CuSCN (NWs)/ZnO as the catalysts are shown in Additional file 1: Figure S6(b). The photocatalytic performance of pristine CuSCN is poorer than that of pristine ZnO nanorods due to smaller specific surface area, lower carrier mobility, and easier recombination of electrons and holes. The photocatalytic performance of CuSCN (NWs) is better than that of CuSCN (3D) owing to the larger specific surface area of the CuSCN nanostructures, as shown in Fig. 4e and f. The photocatalytic performance of CuSCN (3D)/ZnO and CuSCN (NWs)/ZnO is better than that of ZnO due to their heterojunction structure and larger specific surface area. CuSCN (NWs)/ZnO has the better photocatalytic performance than CuSCN (3D)/ZnO due to the smaller and better-distributed ZnO nanorods grown on CuSCN nanostructure and the consequent larger specific surface area. In the ZnO/CuSCN system, ZnO/CuSCN architecture has the best photocatalytic performance among ZnO, CuSCN, ZnO/CuSCN, and CuSCN/ZnO as a result of the heterojunction structure, larger specific surface area of the upper material of the heterojunction, and the larger contact area with the MO solution. The ZnO nanorods under CuSCN (3D) structures are partially etched by the CuSCN reaction solution with an erosive pH, and the ZnO nanorods under CuSCN (NWs) structures are mainly etched by the CuSCN reaction solution with a pH of 1.5 only maintaining the outline and a very few remnants of ZnO nanorods as shown in Fig. 4a and b and Additional file 1: Figure S3(a, b). Although the ZnO nanorods under CuSCN (3D) structures are partially etched, the interspace among the nanorods becomes bigger than that among the pristine ZnO nanorods with the consequent larger specific surface area and is clearer and neater than the ZnO nanorods under CuSCN (NWs) structures with almost complete etching. So, the photocatalytic performance of ZnO/CuSCN (3D) is better than that of ZnO/CuSCN (NWs). The influence on nanostructure morphology and orientation of the heterojunction are discussed, and both can affect the photocatalytic performance in the ZnO/CuSCN system. In summary, ZnO/CuSCN (3D) has the best photocatalytic performance in the ZnO/CuSCN system.

Additional file 1: Figure S6(c) shows the concentration changes of MO with ZnO, NiO (1 min), NiO (10 min), ZnO/NiO (1 min), ZnO/NiO (10 min), NiO (1 min)/ZnO, and NiO (10 min)/ZnO as the catalyst. The photocatalytic performance of pristine NiO (10 min) is poorer than that of pristine ZnO nanorods due to the bigger nanostructure, the consequent smaller specific surface area, lower carrier mobility, and easier recombination of electrons and holes. The photocatalytic performance of NiO (1 min) is better than NiO (10 min) and ZnO owing to the much larger specific surface area of the NiO nanostructures, as shown in Fig. 5e and f. The photocatalytic performance of NiO (10 min)/ZnO is poorer than that of ZnO as a result of the even bigger nanostructure of the upper layer NiO (10 min) and the smaller specific surface area. As shown in Fig. 5c, ZnO nanorods are partially exposed growing through the meshwork of NiO (1 min) and the remnant part of the ZnO nanorods are remained in the meshwork. NiO (1 min) nanostructures are the multi-layer interspersed meshwork uniformly distributed on the ITO glass with much higher specific surface area, as shown in Fig. 5e. So, NiO (1 min)/ZnO has a little better photocatalytic performance than ZnO and a lower photocatalytic action than NiO (1 min). The photocatalytic performance of ZnO/NiO (1 min) and ZnO/NiO (10 min) is better than others due to their heterojunction structure and larger specific surface area. ZnO/NiO(1 min) architecture has the best photocatalytic performance in the ZnO/NiO system as a result of the heterojunction structure, extremely higher specific surface area of the upper material in the heterojunction, and the consequent larger contact area with the MO solution. The influence on reaction time and orientation of the heterojunction are discussed and both will give an effect on the photocatalytic performance in the ZnO/NiO system. In summary, ZnO/NiO (1 min) has the best photocatalytic performance in the ZnO/NiO system.

**Fig. 6:**
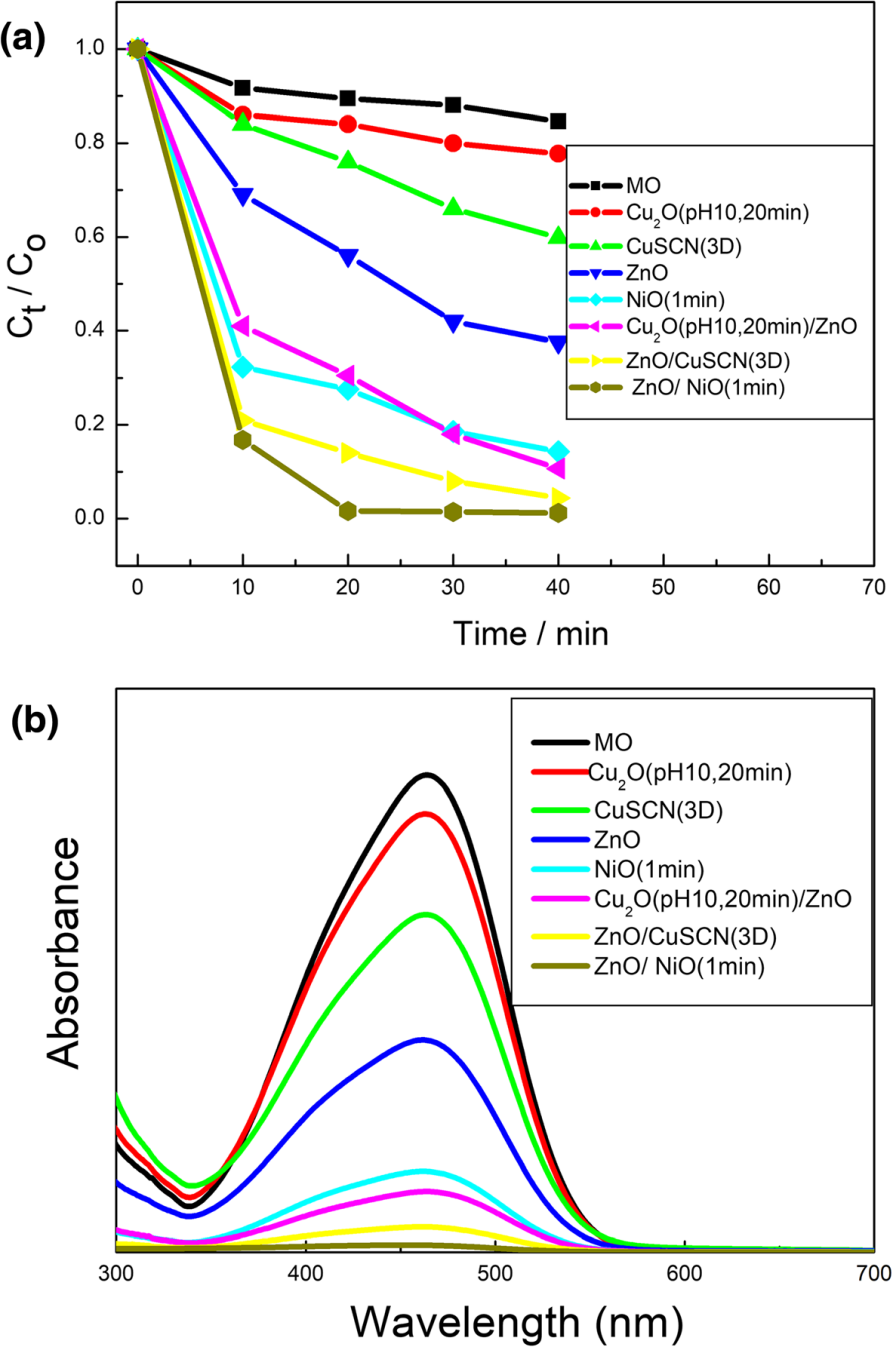
**a** The relative concentration (C_t_/C_0_) of MO versus time under light irradiation in the absence and presence of various photocatalysts: ZnO, Cu_2_O (pH 10, 20 min), CuSCN (3D), NiO (1 min), Cu_2_O (pH 10, 20 min)/ZnO, ZnO/CuSCN (3D), and ZnO/NiO(1 min); **b** The UV-vis absorption spectra of MO aqueous solution with different photocatalysts: ZnO, Cu_2_O (pH 10, 20 min), CuSCN (3D), NiO (1 min), Cu_2_O (pH 10, 20 min)/ZnO, ZnO/CuSCN (3D), and ZnO/NiO(1 min)

Figure 6a and b show the concentration changes of MO and the UV-vis absorption spectra of MO aqueous solution with ZnO, Cu_2_O (pH 10, 20 min), CuSCN (3D), NiO (1 min), Cu_2_O (pH 10, 20 min)/ZnO, ZnO/CuSCN (3D), and ZnO/NiO (1 min) as the catalysts. Among the four semiconductor nanostructures ZnO, Cu_2_O (pH 10, 20 min), CuSCN (3D), and NiO (1 min), NiO has the most excellent photocatalytic performance owing to the multi-layer interspersed meshwork uniformly distributed and the consequent extremely higher specific surface area. ZnO has the bigger mobility and bigger specific surface area than Cu_2_O and CuSCN so that ZnO has the better photocatalytic performance. Cu_2_O has the better photocatalytic performance than CuSCN due to the bigger specific surface area. ZnO/NiO (1 min) heterostructure has the most excellent photocatalytic performance among all the heterostructures based on n-type ZnO and p-type Cu_2_O, CuSCN, and NiO. It is owing to more charge transfer caused by heterojunction structure, more photo-generated carrier as a result of higher specific surface area caused by the meshwork nanostructure of the upper NiO directly contacting to MO, and less carrier recombination caused by more compact contact of NiO/ZnO than Cu_2_O/ZnO and CuSCN/ZnO, as shown in Additional file 1: Figure S2(c), Figure S3(a), and Figure S4(a). In summary, NiO is the most suitable material for photocatalytic degradation of MO among the four semiconductor nanostructures of ZnO, Cu_2_O, CuSCN, and NiO. The photocatalytic performance of the semiconductor can be affected both by the mobility and the specific surface caused by the nanostructure. ZnO/NiO (1 min) heterostructure has the most excellent photocatalytic performance among all the architectures based on ZnO, Cu_2_O, CuSCN, and NiO. Influencing factor on the photocatalytic performance of all these architectures can be summarized as the inherent mobility of the material, the heterojunction architecture, and the morphology of nanostructure. The scheme of the photocatalysis mechanism using heterostructure photocatalyst is shown in Additional file 1: Figure S7. The mechanisms for improved photocatalytic properties are demonstrated in Additional file 1. To further assess the photocatalytic activity, we have compared the degradation ability of our best heterojunction in every system with other nanohybrids in Table 2. Compared with other catalysts, ZnO/NiO (1 min) demonstrated the best photocatalytic performance.

**Table 2:** The degradation rate in the same time (40 min) under the action of different catalysts

Sample	Dye	Dye degradation (%)	Reference
ZnO/Cu_2_O	RhB	50	37
ZnO/CNF/NiO	RhB	78	39
ZnO/NiO	RhB	55	39
ZnO/CdS	MO	95	41
ZnO/NiO (1 min)	MO	99.8	This work
ZnO/Cu_2_O (pH 10, 20 min)	MO	99	This work
ZnO/CuSCN (3D)	MO	99.5	This work

## Conclusions

In summary, different heterojunctions based on n-type ZnO nanorods and p-type Cu_2_O, CuSCN, and NiO nanostructures with different orientations are fabricated. All these structures exhibit certain photocatalytic activity for the degradation of MO. Several conclusions can be summarized with analysis of these photocatalytic data as follows: the morphology of nanostructure has significant influence on photocatalytic activity; the photocatalytic activity of heterojunction structure is better than pristine semiconductor except consideration of the influence of the nanostructure morphology; the orientation of the heterojunction has no remarkable influence on photocatalytic activity; NiO has the best photocatalytic activity among the four pristine semiconductor nanostructures ZnO, Cu_2_O, CuSCN, and NiO; and ZnO/NiO (1 min) heterostructure has the most excellent photocatalytic performance among all the architectures. The great enhancement of the photocatalytic activity is obtained using ZnO/NiO (1 min) heterostructure attributed to the heterojunction structure and extremely higher specific surface area. The study on the influence of materials, nanostructure morphology, and orientation in heterostructure on photocatalytic activity can provide a theoretical direction for the photocatalyst research with application in the energy and environment fields, and it can be concluded with a perspective on the future photocatalyst and a bright prospect of these controllable nanohybrid materials.

## Additional File

Additional file 1: **Figure S1.** The cross-sectional SEM image of ZnO nanorods (a), ZnO/Cu_2_O (pH 12, 20 min) heterojunction (b), Cu_2_O (pH 12, 20 min) (c), and Cu_2_O (pH 12, 20 min)/ZnO heterojunction (d). **Figure S2.** The cross-sectional SEM image of ZnO/Cu_2_O (pH 10, 20 min) heterojunction (a), ZnO/Cu_2_O (pH 10, 40 min) heterojunction (b), Cu_2_O (pH 10, 20 min)/ZnO heterojunction (c), Cu_2_O (pH 10, 40 min)/ZnO heterojunction (d), Cu_2_O (pH 10, 20 min) (e), and Cu_2_O (pH 10, 40 min) (f). **Figure S3.** The cross-sectional SEM image of ZnO/CuSCN (3D) heterojunction (a), ZnO/CuSCN (NWs) heterojunction (b), CuSCN (3D)/ZnO heterojunction (c), CuSCN (NWs)/ZnO heterojunction (d), CuSCN (3D) (e), and CuSCN (NWs) (f). **Figure S4.** The cross-sectional SEM image of ZnO/NiO (1 min) heterojunction (a), ZnO/NiO (10 min) heterojunction (b), NiO (1 min)/ZnO heterojunction (c), NiO (10 min)/ZnO heterojunction (d), NiO (1 min) (e), and NiO (10 min) (f). **Figure S5.** The UV-vis absorption spectra of MO aqueous solution with different photocatalysts: (a) MO degradation in the absence of catalysts; (b) ZnO, Cu_2_O (pH 10, 20 min), Cu_2_O (pH 10, 40 min), Cu_2_O (pH 12, 20 min), ZnO/Cu_2_O (pH 10, 20 min), ZnO/Cu_2_O (pH 10, 40 min), ZnO/Cu_2_O (pH 12, 20 min), Cu_2_O (pH 10, 20 min)/ZnO, Cu_2_O (pH 10, 40 min)/ZnO, and Cu_2_O (pH 12, 20 min)/ZnO; (c) ZnO, CuSCN (3D), CuSCN (NWs), ZnO/CuSCN (3D), ZnO/CuSCN (NWs), CuSCN (3D)/ ZnO, and CuSCN (NWs)/ZnO; (d) ZnO, NiO(1 min), NiO (10 min), ZnO/NiO (1 min), ZnO/NiO (10 min), NiO (1 min)/ZnO, and NiO(10 min)/ZnO. **Figure S6.** The relative concentration (C_t_/C_0_) of MO versus time under light irradiation in the absence and presence of various photocatalysts: (a) ZnO, Cu_2_O (pH 10, 20 min), Cu_2_O (pH 10, 40 min), Cu_2_O (pH 12, 20 min), ZnO/Cu_2_O (pH 10, 20 min), ZnO/Cu_2_O (pH 10, 40 min), ZnO/Cu_2_O (pH 12, 20 min), Cu_2_O (pH 10, 20 min)/ZnO, Cu_2_O (pH 10, 40 min)/ZnO, and Cu_2_O (pH 12, 20 min)/ZnO; (b) ZnO, CuSCN (3D), CuSCN (NWs), ZnO/CuSCN (3D), ZnO/CuSCN (NWs), CuSCN (3D)/ZnO, and CuSCN (NWs)/ZnO; (c) ZnO, NiO (1 min), NiO (10 min), ZnO/NiO (1 min), ZnO/NiO (10 min), NiO (1 min)/ZnO, and NiO (10 min)/ZnO. **Figure S7.** Scheme of the photocatalysis mechanism using heterostructure photocatalyst. (DOCX 2299 kb)

## Abbreviations

1D: One-dimensional
EDTA: Ethylenediaminetetraacetic acid
HMT: Hexamethylenetetramine
MO: Methyl orange
NW: Nanowire
SCE: Saturated calomel electrode
SEM: Scanning electron microscopy
UV-vis DRS: UV-vis diffuse reflectance spectrometry
XRD: X-ray diffraction
