# Supplementary material for: The Influence of Materials, Heterostructure, and Orientation for Nanohybrids on Photocatalytic Activity
Source: Nanoscale Res Lett. 2019 Jan 14;14:20. doi: 10.1186/s11671-019-2851-z (PMC6331350; doi:10.1186/s11671-019-2851-z)
Supplement: Supplementary file 1 — Figure S1. The cross-sectional SEM image of ZnO nanorods (a), ZnO/Cu2O (pH 12, 20 min) heterojunction (b), Cu2O (pH 12, 20 min) (c), and Cu2O (pH 12, 20 min)/ZnO heterojunction (d). Figure S2. The cross-sectional SEM image of ZnO/Cu2O (pH 10, 20 min) heterojunction (a), ZnO/Cu2O (pH 10, 40 min) heterojunction (b), Cu2O (pH 10, 20 min)/ZnO heterojunction (c), Cu2O (pH 10, 40 min)/ZnO heterojunction (d), Cu2O (pH 10, 20 min) (e), and Cu2O (pH 10, 40 min) (f). Figure S3. The cross-sectional SEM image of ZnO/CuSCN (3D) heterojunction (a), ZnO/CuSCN (NWs) heterojunction (b), CuSCN (3D)/ZnO heterojunction (c), CuSCN (NWs)/ZnO heterojunction (d), CuSCN (3D) (e), and CuSCN (NWs) (f). Figure S4. The cross-sectional SEM image of ZnO/NiO (1 min) heterojunction (a), ZnO/NiO (10 min) heterojunction (b), NiO (1 min)/ZnO heterojunction (c), NiO (10 min)/ZnO heterojunction (d), NiO (1 min) (e), and NiO (10 min) (f). Figure S5. The UV-vis absorption spectra of MO aqueous solution with different photocatalysts: (a) MO degradation in the absence of catalysts; (b) ZnO, Cu2O (pH 10, 20 min), Cu2O (pH 10, 40 min), Cu2O (pH 12, 20 min), ZnO/Cu2O (pH 10, 20 min), ZnO/Cu2O (pH 10, 40 min), ZnO/Cu2O (pH 12, 20 min), Cu2O (pH 10, 20 min)/ZnO, Cu2O (pH 10, 40 min)/ZnO, and Cu2O (pH 12, 20 min)/ZnO; (c) ZnO, CuSCN (3D), CuSCN (NWs), ZnO/CuSCN (3D), ZnO/CuSCN (NWs), CuSCN (3D)/ ZnO, and CuSCN (NWs)/ZnO; (d) ZnO, NiO(1 min), NiO (10 min), ZnO/NiO (1 min), ZnO/NiO (10 min), NiO (1 min)/ZnO, and NiO(10 min)/ZnO. Figure S6. The relative concentration (Ct/C0) of MO versus time under light irradiation in the absence and presence of various photocatalysts: (a) ZnO, Cu2O (pH 10, 20 min), Cu2O (pH 10, 40 min), Cu2O (pH 12, 20 min), ZnO/Cu2O (pH 10, 20 min), ZnO/Cu2O (pH 10, 40 min), ZnO/Cu2O (pH 12, 20 min), Cu2O (pH 10, 20 min)/ZnO, Cu2O (pH 10, 40 min)/ZnO, and Cu2O (pH 12, 20 min)/ZnO; (b) ZnO, CuSCN (3D), CuSCN (NWs), ZnO/CuSCN (3D), ZnO/CuSCN (NWs), CuSCN (3D)/ZnO, and CuSCN (NWs)/ZnO; (c) ZnO, NiO [file 11671_2019_2851_MOESM1_ESM.docx]

The Influence of Materials, Heterostructure and Orientation for Nanohybrids on Photocatalytic Activity

Lidan Wang, ^1^ Zisheng Su,^2^ Junsheng Yuan ^1,*^

^1^ *College of Chemical Engineering and Material, Quanzhou Normal University, Quanzhou 362000, Fujian, People's Republic of China*

^2^ *College of Physics and Information Engineering, Quanzhou Normal University, Quanzhou 362000, Fujian, People's Republic of China*

^*^ E-mail: jsyuan2012[@126.com](mailto:agnes777@163.com).

Lidan Wang, agnes777@163.com

Zisheng Su, suzs@ciomp.ac.cn


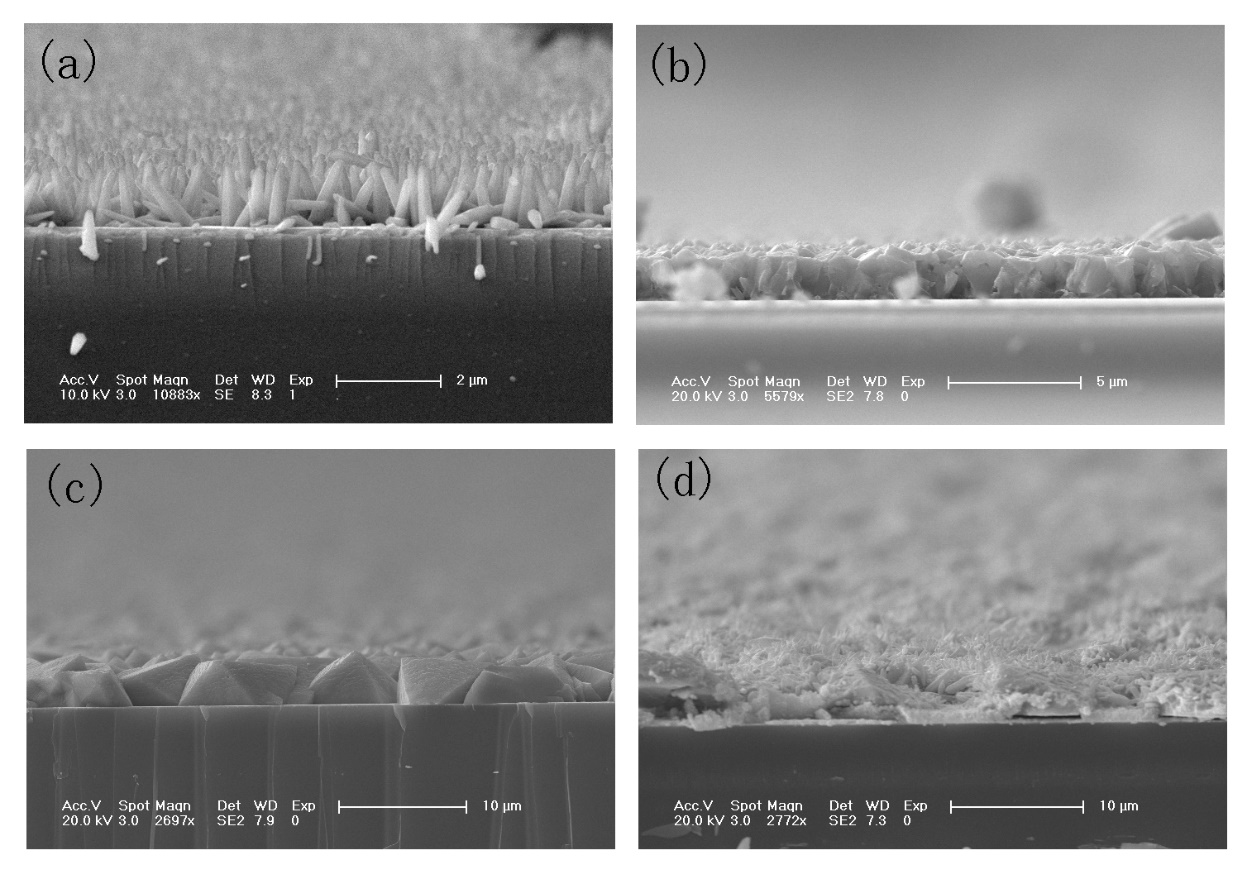


**Fig. S1** The cross-sectional SEM image of ZnO nanorods(a), ZnO/Cu_2_O (pH12, 20 min) heterojunction(b), Cu_2_O (pH12, 20 min) (c) and Cu_2_O (pH12, 20 min)/ZnO heterojunction(d).


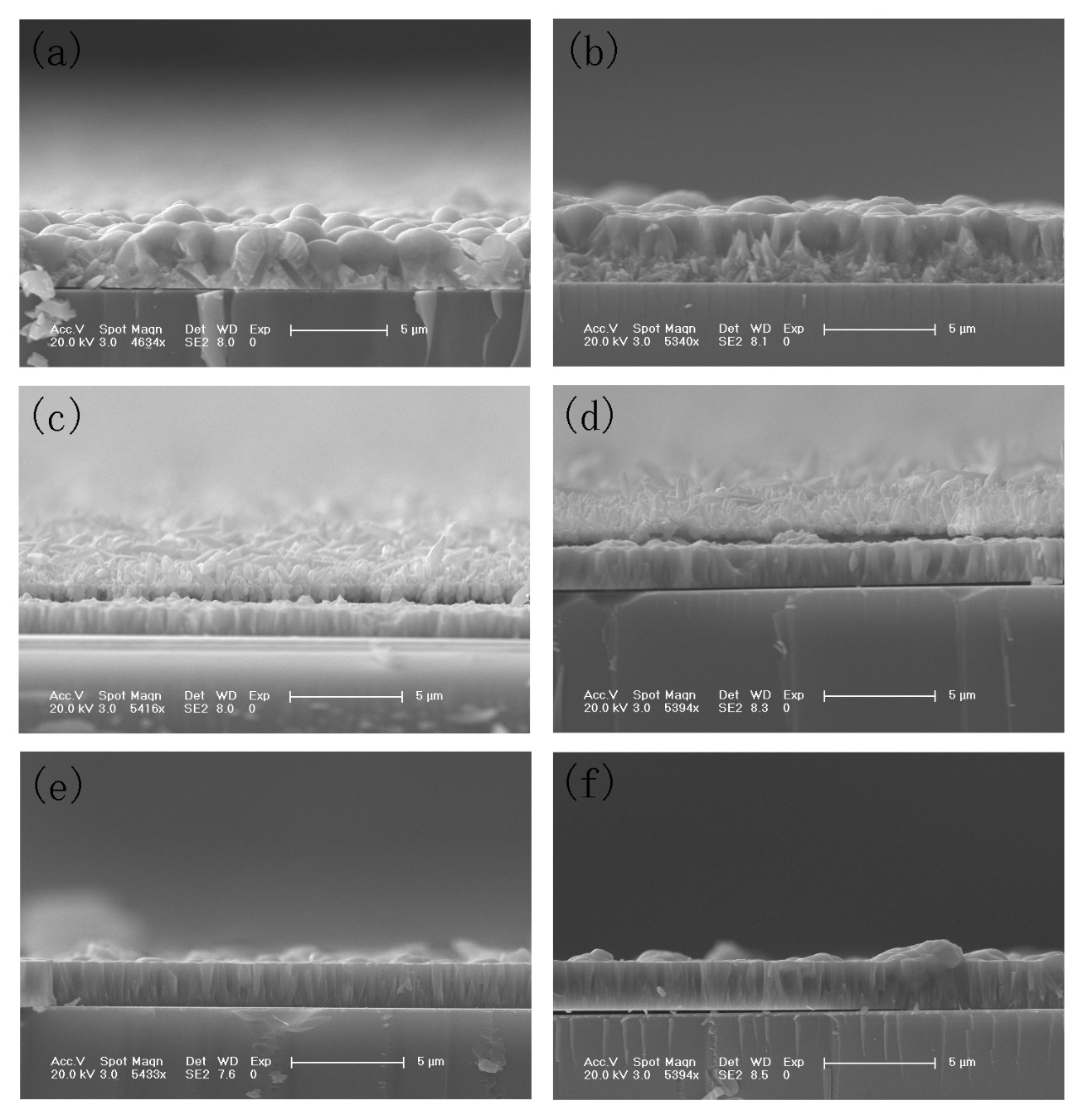


**Fig. S2** The cross-sectional SEM image of ZnO/Cu_2_O (pH10, 20 min) heterojunction(a), ZnO/Cu_2_O (pH10, 40 min) heterojunction(b), Cu_2_O (pH10, 20 min)/ZnO heterojunction(c), Cu_2_O (pH10, 40 min)/ZnO heterojunction(d), Cu_2_O (pH10, 20 min) (e) and Cu_2_O (pH10, 40 min) (f).


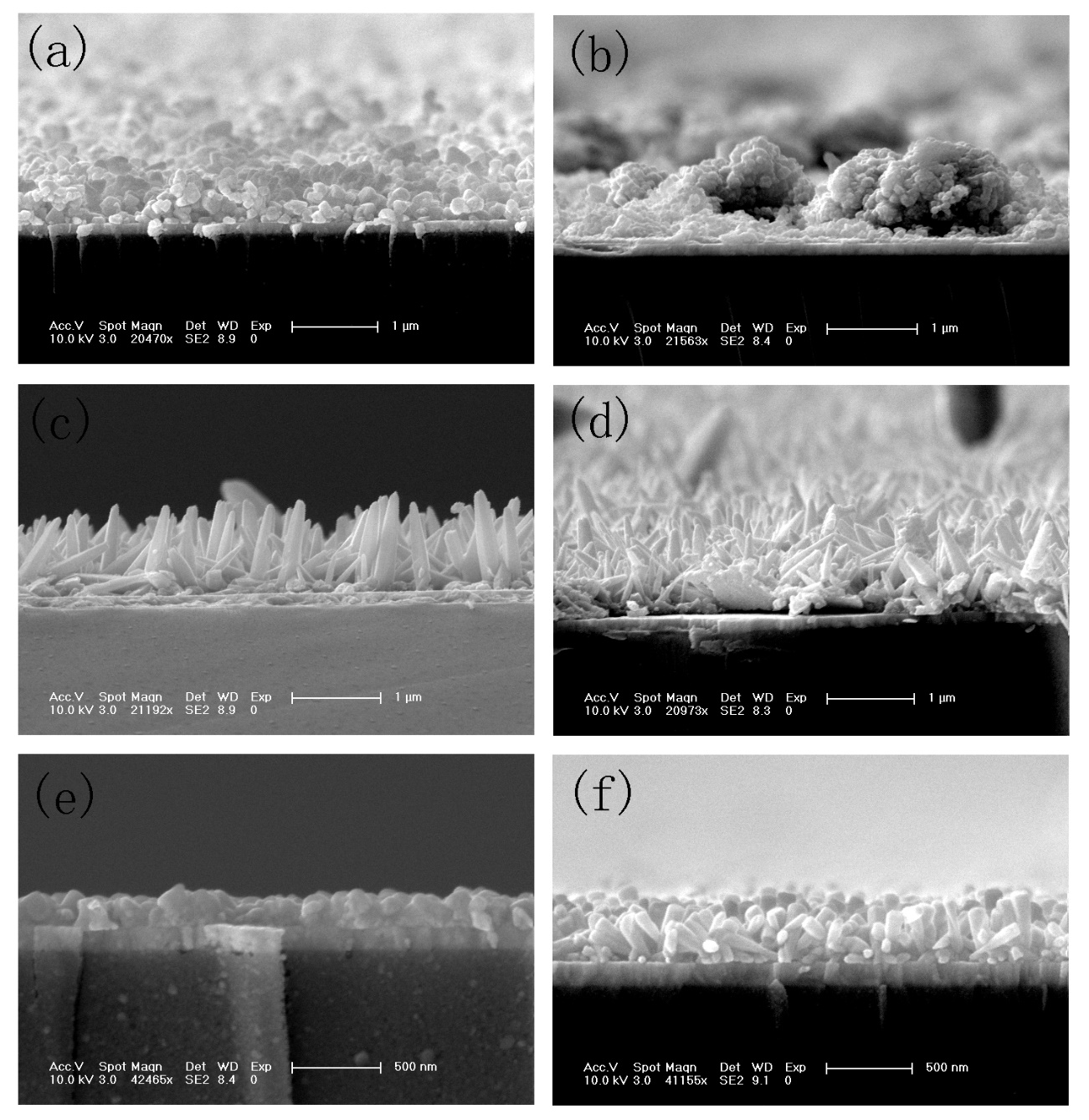


**Fig. S3** The cross-sectional SEM image of ZnO/ CuSCN (3D) heterojunction(a), ZnO/ CuSCN (NWs) heterojunction(b), CuSCN (3D)/ZnO heterojunction(c), CuSCN (NWs)/ZnO heterojunction(d), CuSCN (3D)(e) and CuSCN (NWs)(f).


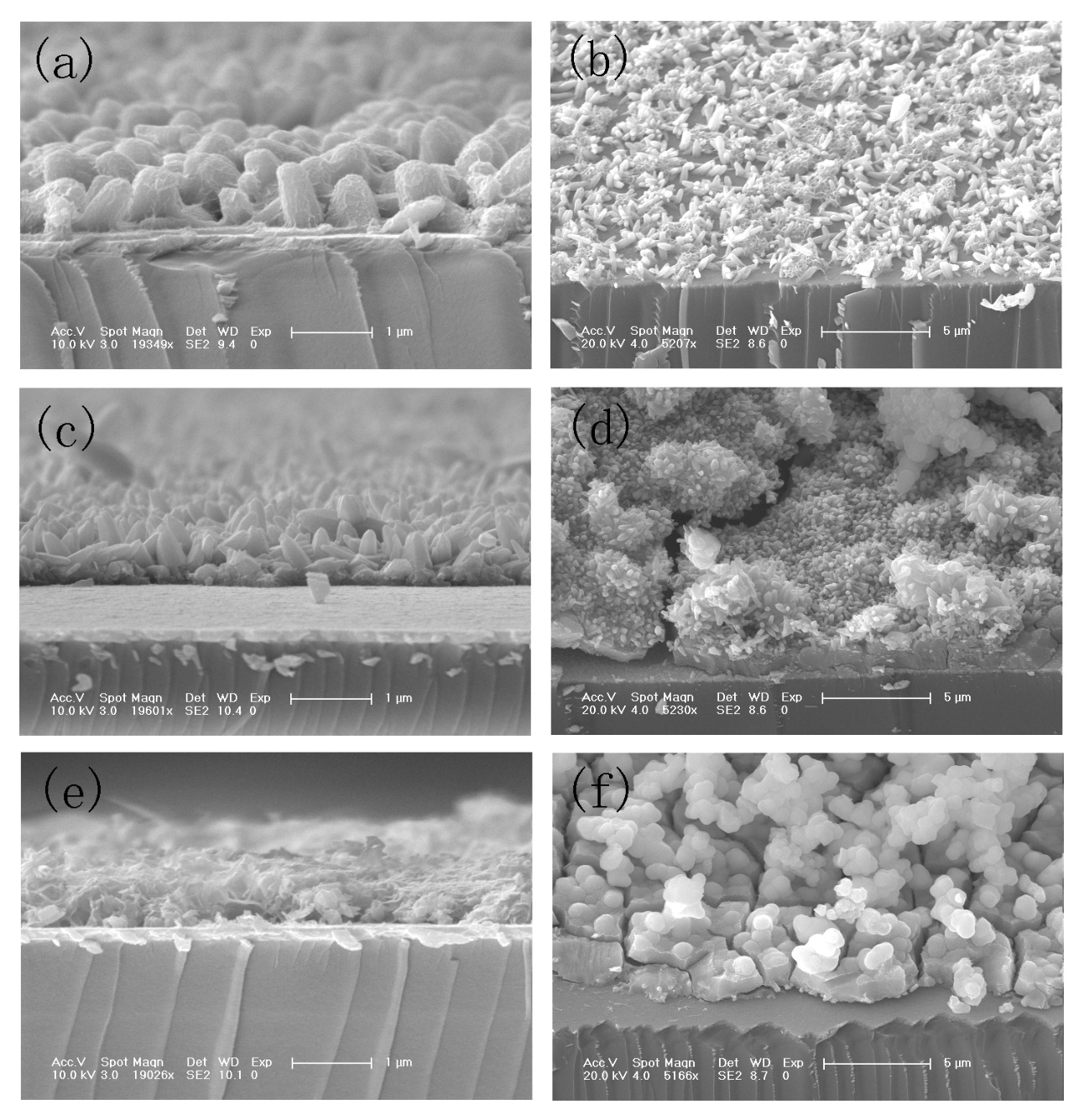


**Fig. S4** The cross-sectional SEM image of ZnO/NiO (1 min) heterojunction(a), ZnO/NiO (10 min) heterojunction(b), NiO (1 min)/ZnO heterojunction(c), NiO (10 min)/ZnO heterojunction(d), NiO (1min) (e), and NiO (10 min) (f).





**Fig. S5** The UV-vis absorption spectra of MO aqueous solution with different photocatalysts: (a) MO degradation in the absence of catalysts; (b)ZnO, Cu_2_O(pH10, 20 min), Cu_2_O(pH10, 40 min), Cu_2_O(pH12, 20 min), ZnO/Cu_2_O(pH10, 20 min), ZnO/Cu_2_O(pH10, 40 min), ZnO/Cu_2_O(pH12, 20 min), Cu_2_O(pH10, 20 min)/ZnO, Cu_2_O(pH10, 40 min)/ZnO and Cu_2_O(pH12, 20 min)/ZnO; (c) ZnO, CuSCN(3D), CuSCN(NWs), ZnO/CuSCN(3D), ZnO/CuSCN(NWs), CuSCN(3D)/ ZnO and CuSCN(NWs)/ ZnO; (d) ZnO, NiO(1 min), NiO(10 min), ZnO/NiO(1 min), ZnO/NiO(10 min), NiO(1 min)/ZnO and NiO(10 min)/ZnO.





**Fig. S6** The relative concentration (C_t_/C_0_) of MO versus time under light irradiation in the absence and presence of various photocatalysts: (a) ZnO, Cu_2_O(pH10, 20 min), Cu_2_O(pH10, 40 min), Cu_2_O(pH12, 20 min), ZnO/Cu_2_O(pH10, 20 min), ZnO/Cu_2_O(pH10, 40 min), ZnO/Cu_2_O(pH12, 20 min), Cu_2_O(pH10, 20 min)/ZnO, Cu_2_O(pH10, 40 min)/ZnO and Cu_2_O(pH12, 20 min)/ZnO; (b) ZnO, CuSCN(3D), CuSCN(NWs), ZnO/CuSCN(3D), ZnO/CuSCN(NWs), CuSCN(3D)/ ZnO and CuSCN(NWs)/ ZnO; (c) ZnO, NiO(1 min), NiO(10 min), ZnO/NiO(1 min), ZnO/NiO(10 min), NiO(1 min)/ZnO and NiO(10 min)/ZnO.





**Fig. S7** Scheme of the photocatalysis mechanism using heterostructure photocatalyst.

**Mechanisms for improved photocatalytic properties.**

The energy band graph of ZnO/ Cu_2_O/ CuSCN/ NiO is shown in Figure S7. The p-n junction structure of n-type ZnO and p-type Cu_2_O (or CuSCN or NiO) can promotes efficient separation of electron-hole pairs and prevents their recombination. When the p-n junction structures are irradiated with light, the electrons in the conduction band (CB) of p-type material can migrate into the ZnO CB, while the holes transfer from the valence band (VB) of ZnO to the VB of p-type material in the opposite direction. The internal field at the p-n junction interface facilitates the formation of charge transfer states and the spatial separation of the photo-generated carriers. After the separation, the electrons accumulated on the surface of ZnO can react with water soluble O_2_, and then form superoxide radicals (·O_2_^-^) and hydroxyl radicals (·OH) to oxidize the organic dye. The holes in p-type material can interact with H_2_O to form ·OH with high activity. The ·OH plays an important role in the decomposition of organic dyes. The mechanism for the photodegradation of MO by the p-n junction can be summarized as follows:

(ZnO/p-type material) + hν → ZnO(e^-^)/ p-type material(h^+^) (1)

e^-^ + O_2_ →•O_2_^-^ (2)

•O_2_^-^ + H_2_O → OH^-^ + •HO_2_ (3)

•HO_2_ + H_2_O → H_2_O_2_ + •OH (4)

H_2_O_2_ → 2•OH (5) h^+^ + H_2_O → H^+^ + •OH (6)

•OH + MO → degradation products (7)
